# Arthroscopic-assisted uniportal spinal surgery for the treatment of rare T10/T11 thoracic disc herniation: a case report

**DOI:** 10.3389/fsurg.2026.1918397

**Published:** 2026-09-01

**Authors:** De Meng, GuoFang Meng, ShenRong Jiang, Yuan Zhong

**Affiliations:** 1 Department of Spine Surgery, The Second People's Hospital of Pingnan County, Guigang, China; 2 Department of Orthopedics and Traumatology, Traditional Chinese Medicine Hospital of Pingnan County, Guigang, China; 3 Department of Spine Surgery, The Second Affiliated Hospital of Guangxi Medical University, Nanning, China

**Keywords:** arthroscopic-assisted uni-portal spinal surgery, case report, spinal canal decompression, thoracic disc herniation, treatment

## Abstract

**Background:**

Thoracic disc herniation (TDH) is rare, with an estimated incidence of 1 per 1,000,000 individuals. Surgical treatment is indicated for those suffering from myelopathy and/or intractable neurological deficit. Open surgical approaches including transthoracic and retropleural access carry substantial perioperative risks. Contemporary minimally invasive endoscopic techniques reduce tissue dissection and manipulation of the thecal sac, yet they are associated with a steep learning curve and limited intraoperative maneuverability. This study reports the case of a 76-year-old male with TDH who underwent decompression using arthroscopic-assisted uniportal spinal surgery (AUSS) for the first time, achieving effective short-term therapeutic outcomes.

**Case presentation:**

A 76-year-old male presented with progressive numbness and weakness in both lower extremities persisting for over one month. Computed tomography (CT) and magnetic resonance imaging (MRI) revealed T10/T11 disc herniation with severe compression of the thoracic spinal cord. The herniated nucleus pulposus was excised via AUSS, thereby achieving decompression of the thoracic spinal cord. The patient recovered uneventfully without complications, and postoperative CT and MRI confirmed complete spinal cord decompression.

**Conclusion:**

The application of the AUSS technique for the treatment of TDH is feasible. The AUSS approach offers several advantages, including minimal invasiveness, reduced surgical trauma, less blood loss, faster recovery, and greater operational flexibility.

## 1 Introduction

Thoracic disc herniation (TDH) is a rare condition. TDH affects 0.001%–0.1% of the general population, accounting for 0.1%–3% of all disc herniation cases (1). In the absence of symptomatic myelopathy, non-surgical treatment is suitable for most cases (2, 3). Surgery is indicated if conservative treatment fails, or neurological symptoms newly emerge or worsen. Even without clinical nerve signs, MRI spinal cord lesions (T1 hypointensity, intramedullary T2 hyperintensity) are also surgical indications (4). Early intervention is recommended to halt disease progression and prevent irreversible spinal cord damage.

Clinically, most TDH patients undergo posterior/posterolateral thoracic decompression or transthoracic anterior discectomy. These traditional open procedures have large incisions and severe trauma, raising risks of intercostal neuralgia, lung collapse, dural tear, cerebrospinal fluid leakage and poor wound healing (4–6). With the development of minimally invasive spinal surgery, microdiscectomy and endoscopic surgery can treat symptomatic TDH as reported, yet they carry steep learning curves and elevated risks of spinal cord injury, dural tear and cerebrospinal fluid leakage, with limited maneuverability, surgical vision and decompression range (7–10).

Professor En Song first proposed decompression via arthroscope-assisted single-port spinal surgery (AUSS) in 2021 (11). Combining the strengths of uniaxial and biaxial endoscopy (12, 13), this technique has the following features: i) Continuous irrigation with aqueous medium enables full intraoperative visualization; ii) Unlike unilateral biportal endoscopy (UBE), it integrates two working portals into a single portal. iii) Different from uniportal coaxial endoscopy, in which the endoscope and instruments are separated, AUSS allows the endoscope and surgical instruments to operate within the same single portal on one identical plane. iv) It delivers convenient access and flexible manipulation, and realizes endoscopic minimally invasive modification of conventional open spinal surgery (14, 15).

AUSS has been proven safe and effective for the treatment of lumbar disorders (11). To the best of our knowledge, there have been no relevant studies specifically reporting the use of AUSS for the treatment of thoracic disc herniation. This study presents the case of a patient with TDH who underwent decompression using AUSS, achieving effective short-term outcomes.

## 2 Case presentation

A 76-year-old male patient was admitted to the hospital in March 2026. More than one month prior to admission, he developed numbness and pain in the bilateral inguinal regions and anteromedial aspects of both thighs accompanied by bilateral lower limb weakness without obvious inducement. Conservative treatment failed to alleviate his symptoms, which progressively worsened. The patient had a medical history of hypertension and type 2 diabetes mellitus. Physical examination revealed hypoesthesia in the bilateral inguinal regions and the anteromedial aspects of both thighs. Muscle strength grade 3/5 was noted in the left iliopsoas, tibialis anterior, triceps surae and dorsiflexors. The right iliopsoas, biceps femoris, tibialis anterior, triceps surae and dorsiflexors were graded 3-/5. All other muscle groups exhibited grade 5/5 strength. Hyperreflexia of the bilateral patellar tendons, and positive Babinski signs bilaterally. His neurological function score was 12 according to the modified Japanese Orthopaedic Association (mJOA) scoring system.

## 3 Diagnosis and surgical treatment

**Figure 1:**
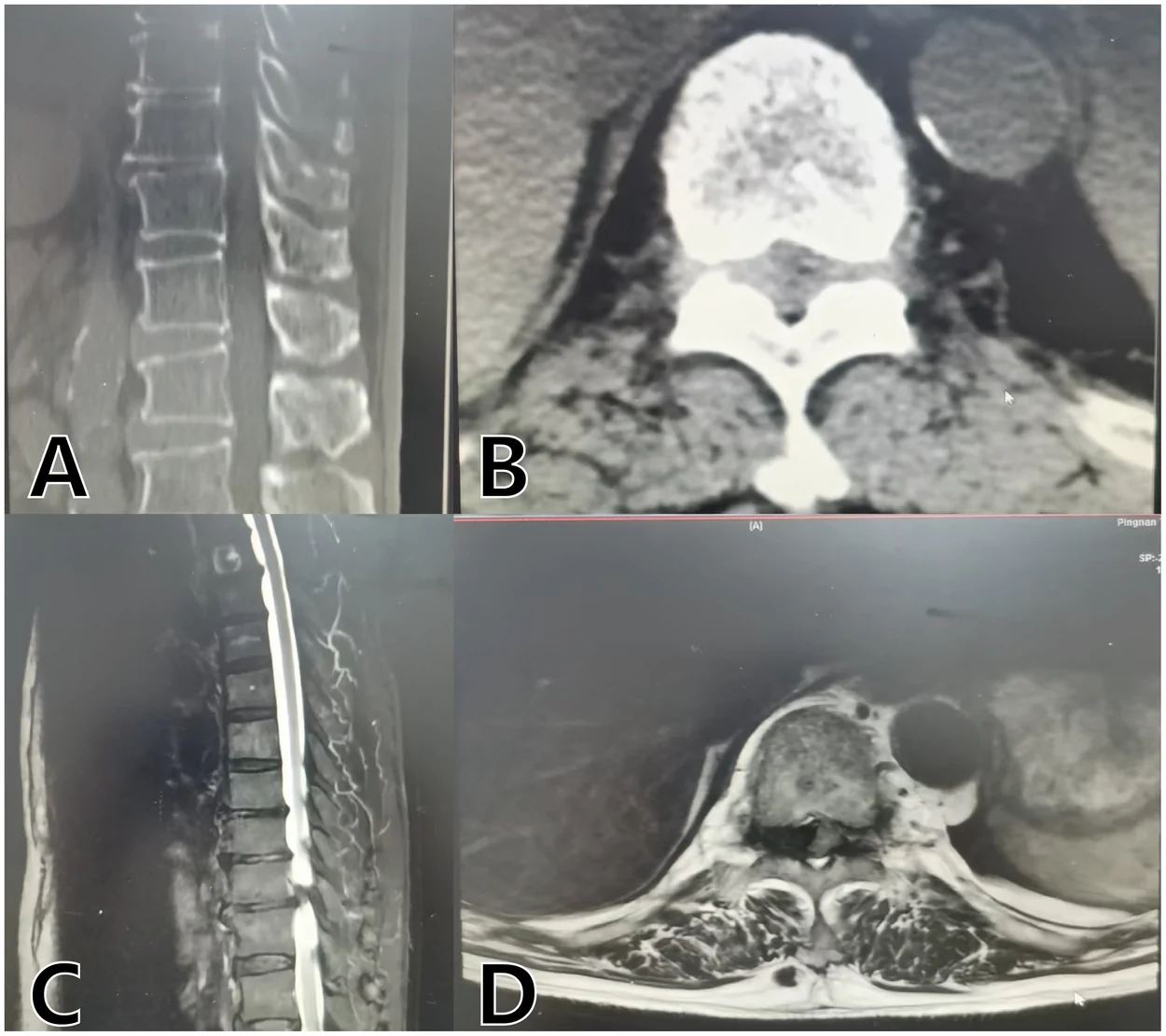
Preoperative images. **(A,B)** CT image taken at the T10/11 level show thoracic disc herniation. **(C,D)** MRI scan taken at the T10/11 level show severe spinal cord compression. CT, computed tomography; MRI, magnetic resonance imaging.

Clinical and radiographic evaluation by three senior spine surgeons and three radiologists revealed computed tomography (CT) demonstrated a T10/T11 TDH (Figures 1A,B). Magnetic resonance imaging (MRI) revealed severe compression of the thoracic spinal cord (Figures 1C,D). The patient elected to undergo surgical decompression and provided informed consent for both the procedure and the publication of case-related data. Following detailed preoperative planning, AUSS was selected for thoracic intradiscal nucleotomy and spinal cord decompression.

## 4 Surgical technique

### 4.1 Localization method

**Figure 2:**
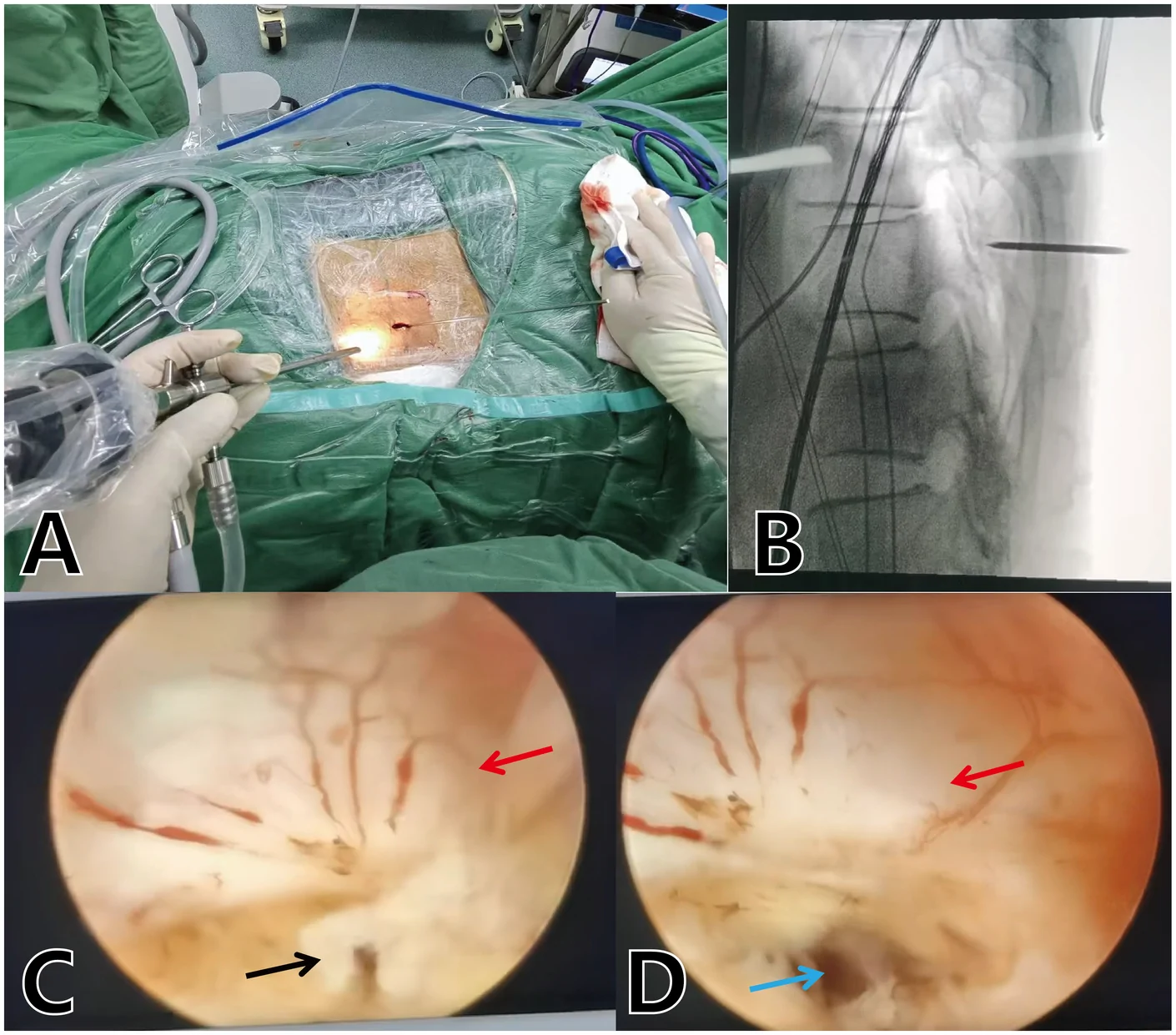
Intraoperative images. **(A)** The entry point for endoscopic instruments. **(B)** Entry point confirmed by fluoroscopy. **(C)** Under arthroscopic visualization, the herniated intervertebral disc was visible. **(D)** Full thoracic thecal sac decompression & re-expansion. Red arrows: dura, black arrows: herniated intervertebral disc, blue arrows: tear of the annulus fibrosus.

Following anesthesia induction, the patient lay prone on an abdomen-free frame with a horseshoe headrest for slight spinal flexion. C-arm fluoroscopy identified the pathological T10/T11 level. Two surface landmarks were drawn: the vertical projection of T10/T11 disc space perpendicular to spinous processes, and a line parallel to spinous processes connecting midpoints of right T10 and T11 pedicles. A 1.8 cm longitudinal incision perpendicular to the intervertebral space was made at their intersection (Figure 2A). A localization guide needle was inserted through the incision, and C-arm fluoroscopy confirmed that the tip of the guide needle had reached the inferior margin of the right T10 lamina (Figure 2B).

### 4.2 Exposure

A dilator was then inserted through the localization guide needle. Serial dilators dilated and separated muscles via the incision. A T-shaped dissector cleared soft tissues on the lamina, followed by arthroscope placement linked to gravity saline irrigation. Radiofrequency devices dissected soft tissues for complete pre- and intraoperative hemostasis, revealing the right T10 spinous process-lamina junction, medial facet margin and superior edge of the right T11 lamina.

### 4.3 Spinal canal enlargement

Boneat the inferior lamina was thinned with a high-speed burr via the circular grinding method. Subsequently, Kerrison rongeurs were used to resect portions of the lamina and facet bone to expose the ligamentum flavum overlying the herniated disc. The ligamentum flavum was dissected using a nerve dissector. A small tear was then created in the ligamentum flavum with Kerrison rongeurs, following which the ligamentum flavum overlying the herniated disc was resected along this tear. Radiofrequency devices and nerve dissectors gently separated and retained epidural fat, released adhesions in front of the thecal sac, and fully exposed the herniated nucleus pulposus and thecal sac (Figure 2C).

### 4.4 Resecting the herniated disc and spinal canal decompression

Under endoscopic visualization, pituitary rongeurs and nucleus pulposus scissors of various sizes and angles were employed to remove the protruded intervertebral disc tissue (Figure 2C). The spinal cord and nerve roots were re-explored and released to ensure their relaxation and the absence of dural sac compression (Figure 2D). Bipolar radiofrequency ablation was then used to assist in T10/11 disc decompression, annulus fibrosus tear shrinkage, and capsulorrhaphy.

## 5 Outcomes

**Figure 3:**
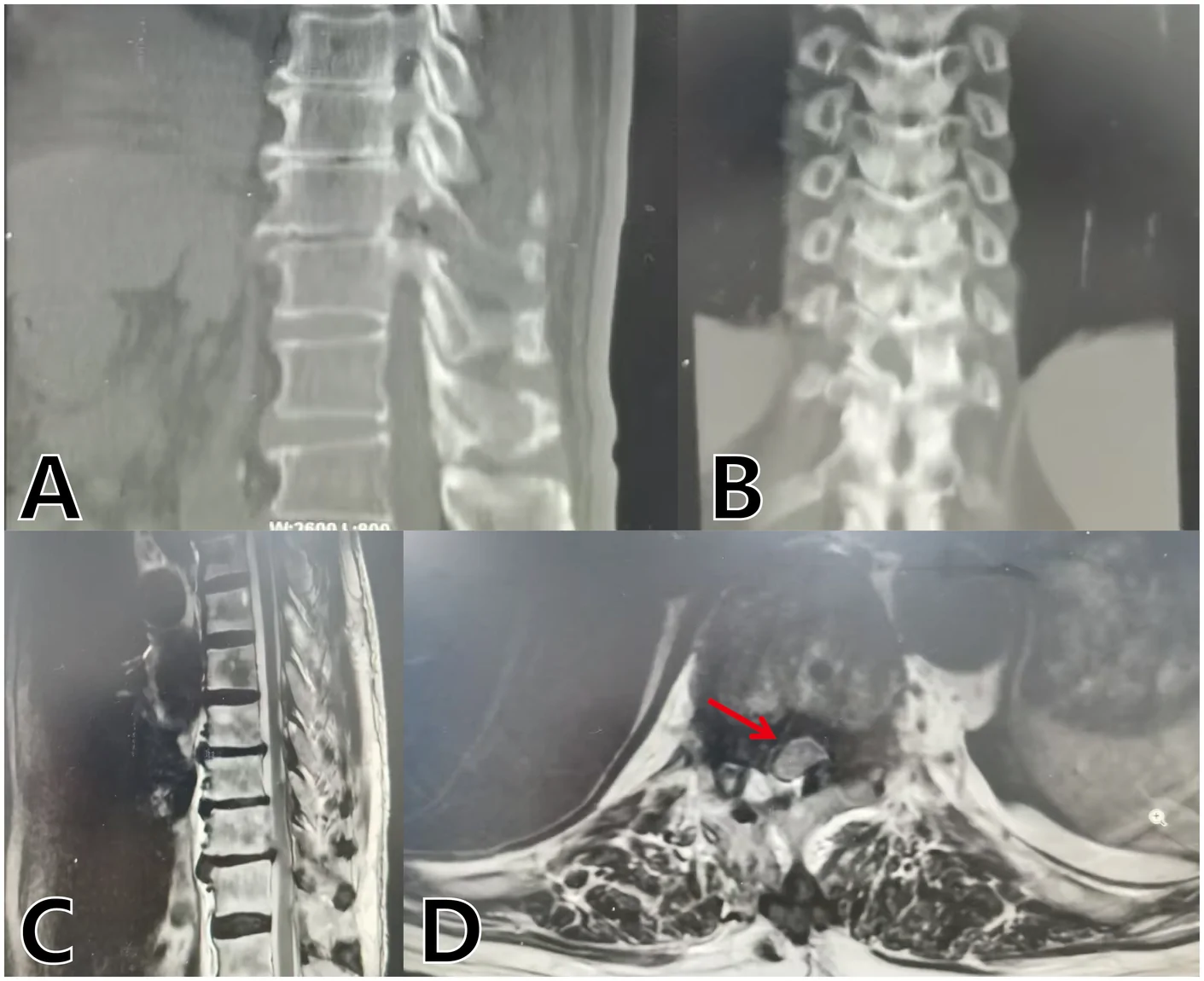
Postoperative day 3 images. **(A,B)** CT image taken at the T10/11 level confirm under arthroscopic visualization, laminar fenestration and decompression were performed. **(C,D)** MRI scan taken at the T10/11 level confirm thorough decompression of the spinal cord. CT, computed tomography; MRI, magnetic resonance imaging.

**Figure 4:**
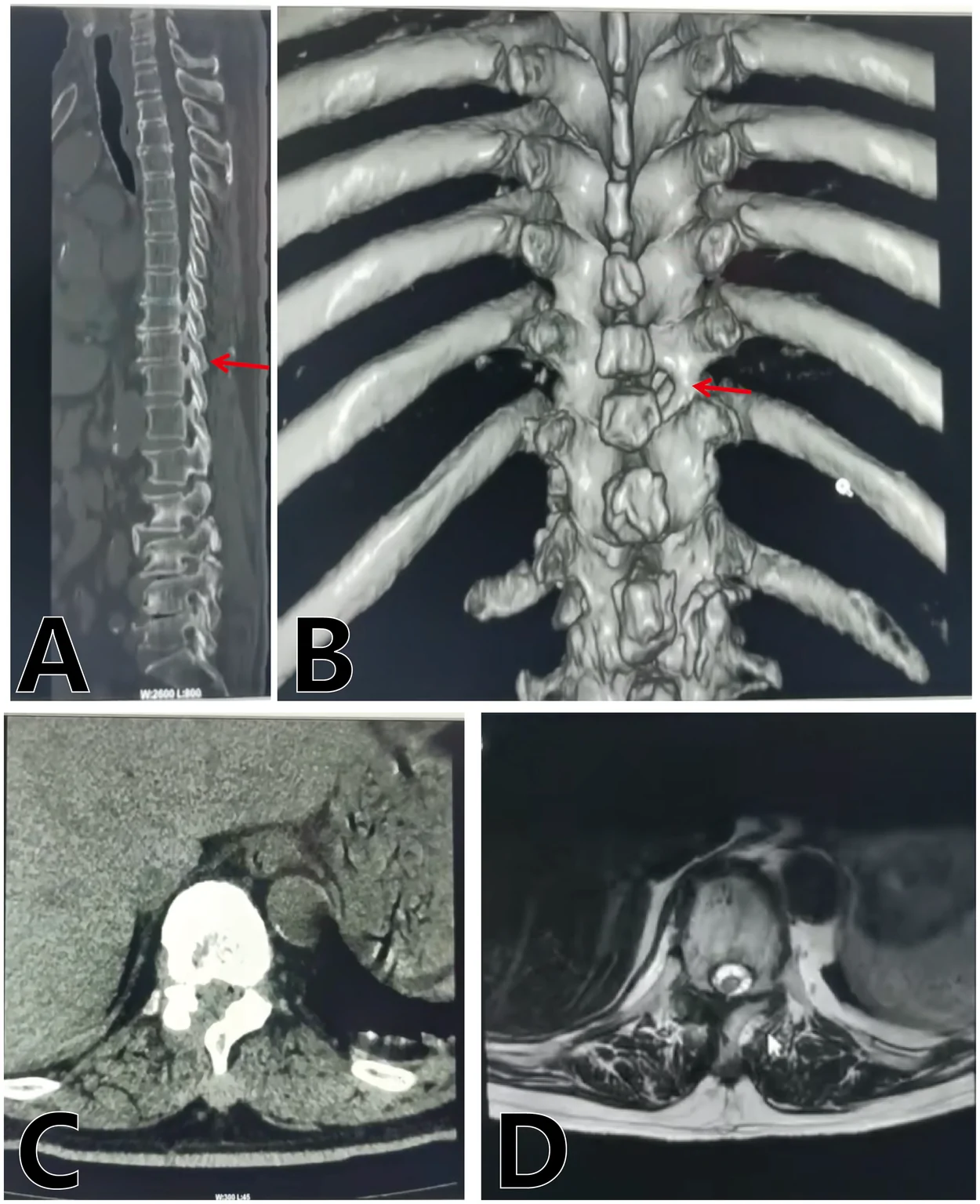
5-months post-operation images. **(A–C)** CT showed no evidence of postoperative vertebral instability. **(D)** MRI demonstrated recovery of spinal cord distension. Red arrows: herniated segment. CT, computed tomography; MRI, magnetic resonance imaging.

Postoperative numbness was relieved compared with preoperative conditions, and pain completely disappeared. On postoperative day 3, bilateral lower limb muscle strength reached grade 4/5, and the mJOA score was 13. CT and MRI scans obtained on postoperative day 3 confirmed complete resolution of ventral compression at the T10/11 level and thorough decompression of the thoracic spinal cord (Figures 3A–D). During the 5-month follow-up period, no clinical signs of spinal instability were identified on CT (Figures 4A–C). MRI revealed spinal cord re-expansion compared with the preoperative status (Figure 4D). During the 5-month follow-up period, the patient was able to ambulate with the aid of a walker, and the mJOA score improved to 14. These findings indicate a satisfactory surgical outcome.

## 6 Discussion

### 6.1 Symptomatic TDH occurs infrequently

Symptomatic TDH constitutes less than 1% of all disc herniation cases and is significantly less common than cervical or lumbar disc herniations (16). Approximately 75% of TDH cases occur below the T7-T8 level, which is the most susceptible to degeneration. This susceptibility may be attributed to the slightly greater mobility and relative weakness of the posterior longitudinal ligament at this level (16). Most TDHs are located centrally or posterolaterally (17). The present case report describes a 76-year-old male patient with a posterolateral herniation of the T10/11 disc.

### 6.2 Challenges in treating TDH

Thoracic spine surgery is technically challenging for the following reasons: i) Rib attachments hinder exposure of the thoracic spinal canal; ii) Physiologic thoracic kyphosis compresses the dural sac against the posterior edge of the intervertebral disc; iii) Denticulate ligaments restrict spinal cord mobility; iv) The spinal cord occupies most of the canal with narrow surrounding space; v) Several thoracic segments have inadequate spinal cord blood supply (4).

Conventional TDH surgical approaches include posterior laminectomy, posterolateral transpedicular/transforaminal routes, lateral costotransversectomy/extrapleural accesses, transthoracic techniques (thoracotomy, mini-thoracotomy, thoracoscopy) and anterior sternotomy (7). Although the transthoracic approach provides adequate visualization and decompression of the ventral dural sac, thoracotomy significantly compromises pulmonary function and necessitates a prolonged postoperative recovery period. The open posterolateral transpedicular approach allows lateral access to the disc, reducing the risk of spinal cord retraction. Nevertheless, this technique requires partial resection of the pedicle and facet joint, substantially compromising spinal stability and necessitating additional pedicle screw fixation and internal stabilization (4–6). Mixter and Barr first described TDH discectomy via routine posterior laminectomy in 1934 (18), which carried 18%–75% complication rates and up to 50% mortality (19, 20). To address these drawbacks, spine surgeons explored alternative open approaches. Nevertheless, overall complication rates exceeding 25% have been reported in TDH patients undergoing open surgery (21).

Large incisions and severe trauma of open TDH surgery increase risks of spinal instability, intercostal neuralgia, atelectasis, dural tear, CSF leakage and poor wound healing (6, 22). With technical progress, minimally invasive surgery has become the primary treatment for TDH (6, 22). Accumulating evidence confirms minimally invasive surgery yields less tissue dissection, blood loss and epidural fibrosis, suiting elderly and immunocompromised patients (23, 24). However, uniportal endoscopy and microscopy feature steep learning curves, limited vision, tight operative space, inadequate decompression, low efficiency, residual nucleus pulposus and high recurrence risk (25, 26). To address uniportal endoscopy defects, Professor Ren Shiwei reported unilateral biportal endoscopy (UBE) technique for TDH (10). Independent viewing and working portals offer better instrument manipulation and broader vision (10). But studies indicate UBE has a steep short-term learning curve, with intraoperative risks of unstable instrument handling and fluctuating visual field (27, 28).

### 6.3 AUSS advantages and limitations

In 2021, Professor Song En introduced the concept of the AUSS technique (11). As a novel minimally invasive spinal surgery approach, the AUSS technique utilizes a single surgical incision, allowing the surgeon to hold the arthroscope with one hand while operating radiofrequency and surgical instruments with the other (11). The AUSS technique uses standard spinal posterior decompression instruments. It achieves efficient surgery and equivalent decompression results to posterior open surgery, with less trauma, faster recovery and better minimal invasiveness (11). Uniportal endoscopy and UBE have obvious shortcomings including steep learning curves, narrow visual fields, limited intraoperative space, inadequate decompression and low efficiency (25–28). As a modified single-portal technique based on UBE with shared instruments, AUSS provides broad vision and flexible operation space, less tissue damage and clear HD visualization of spinal neural and vascular structures. It supports sufficient decompression and effective hemostasis to lower nerve injury risks (11). Consistent with traditional open fenestration discectomy and using standard posterior decompression devices, AUSS features a gentle learning curve for surgeons familiar with open spinal procedures (11).

The patient was elderly and suffered from comorbidities such as hypertension and diabetes, indicating minimally invasive surgery was preferable. Due to anatomical constraints, operations on the thoracic spinal canal are associated with an elevated risk of spinal cord injury (4). Although the AUSS technique has been extensively used for lumbar spine surgery (11), there have been no published reports regarding its use for thoracic disc herniation. The AUSS technique enables adequate decompression of the stenotic thoracic spinal canal similar to open surgery via a minimally invasive approach, which can expand the space for the spinal cord before removing the herniated disc and lower the risk of spinal cord injury. Therefore, this study represents the first application of the AUSS technique in the treatment of TDH. With the assistance of arthroscopy, we successfully resected the herniated thoracic disc. The patient's clinical symptoms were significantly ameliorated. However, this report has several limitations. First, it is a single-case report, and its clinical representativeness and generalizability of the technique need to be verified by large cohort studies. Second, its potential long-term impacts on spinal stability remain unclear. Finally, short-term follow-up is insufficient to fully evaluate its efficacy. Prospective, multicenter, long-term studies are required for assessment in the future.

## 7 Conclusion

The findings of this case indicate that the application of the AUSS technique for the treatment of TDH is feasible. The AUSS procedure offers several advantages, including minimal invasiveness, faster recovery, reduced tissue trauma, less blood loss, and greater operational flexibility.

## Data Availability

The original contributions presented in the study are included in the article/Supplementary Material, further inquiries can be directed to the corresponding author.

## References

[1] Bouthors C Benzakour A Court C. Surgical treatment of thoracic disc herniation: an overview. Int Orthop. (2019) 43(4):807–16. 10.1007/s00264-018-4224-0 30406842

[2] Alonso F Kasliwal MK. Regression of giant calcified thoracic disk and spinal cord compression following thoracic laminectomy and posterior instrumented fusion. World Neurosurg. (2018) 110:64. 10.1016/j.wneu.2017.10.122 29101074

[3] Cofano F Berjano P Vercelli G Palmieri G Pejrona M Zenga F. Spontaneous regression of calcified thoracic herniations: can Hounsfield-units radiodensity have a predictive value? Eur Spine J. (2020) 29(7):1717–23. 10.1007/s00586-019-06192-x 31664568

[4] Court C Mansour E Bouthors C. Thoracic disc herniation: surgical treatment. Orthop Traumatol Surg Res. (2018) 104(1S):S31–40. 10.1016/j.otsr.2017.04.022 29225115

[5] Baranowska J Baranowska A Baranowski P Rybarczyk M. Surgical treatment of thoracic disc herniation. Pol Merkur Lekarski. (2020) 49(286):267–70. 32827423

[6] Kerezoudis P Rajjoub KR Goncalves S Alvi MA Elminawy M Alamoudi A. Anterior versus posterior approaches for thoracic disc herniation: association with postoperative complications. Clin Neurol Neurosurg. (2018) 167:17–23. 10.1016/j.clineuro.2018.02.009 29428625

[7] Bae J Kim J Lee SH Kim JS. Comparative analysis of transforaminal endoscopic thoracic discectomy and microscopic discectomy for symptomatic thoracic disc herniation. Neurospine. (2022) 19(3):555–62. 10.14245/ns.2244294.147 36203281 PMC9537848

[8] Bae J Chachan S Shin SH Lee SH. Percutaneous endoscopic thoracic discectomy in the upper and midthoracic spine: a technical note. Neurospine. (2019) 16(1):148–53. 10.14245/ns.1836260.130 30943717 PMC6449831

[9] Houra K Saftic R. Transforaminal endoscopic discectomy for large, two level calcified, thoracic disc herniations with 5-year follow-up. Neurospine. (2020) 17(4):954–9. 10.14245/ns.2040090.045 33401876 PMC7788411

[10] Ren S Chang Z Pan J. UBE therapy for dual-segment thoracic disc herniation. Case Rep Med. (2025) 2025:3795148. 10.1155/carm/3795148 41281623 PMC12634169

[11] Wang F Wang R Zhang C Song E Li F. Clinical effects of arthroscopic-assisted uni-portal spinal surgery and unilateral bi-portal endoscopy on unilateral laminotomy for bilateral decompression in patients with lumbar spinal stenosis: a retrospective cohort study. J Orthop Surg Res. (2024) 19(1):167. 10.1186/s13018-024-04621-2 38444008 PMC10916320

[12] Kotheeranurak V Tangdamrongtham T Lin G-X Singhatanadgige W Limthongkul W Yingsakmongkol W. Comparison of full-endoscopic and tubular-based microscopic decompression in patients with lumbar spinal stenosis: a randomized controlled trial. Eur Spine J. (2023) 32(8):2736–47. 10.1007/s00586-023-07678-5 37010607 PMC10068229

[13] Yu Y Jiang Y Xu F Yuan L Mao Y Li C. Percutaneous full-endoscopic uniportal decompression for the treatment of symptomatic idiopathic lumbar spinal epidural lipomatosis: technical note. Front Surg. (2022) 9:894662. 10.3389/fsurg.2022.894662 36147697 PMC9485546

[14] Junjie L Jiheng Y Jun L Haixiong L Haifeng Y. Comparison of unilateral biportal endoscopy decompression and microscopic decompression effectiveness in lumbar spinal stenosis treatment: a systematic review and meta-analysis. Asian Spine J. (2023) 17(2):418–30. 10.31616/asj.2021.0527 36740930 PMC10151631

[15] Kang SS Lee SC Kim SK. A novel percutaneous biportal endoscopic technique for symptomatic spinal epidural lipomatosis: technical note and case presentations. World Neurosurg. (2019) 129:49–54. 10.1016/j.wneu.2019.05.214 31154106

[16] Kasliwal MK Deutsch H. Minimally invasive retropleural approach for central thoracic disc herniation. Minim Invasive Neurosurg. (2011) 54(4):167–71. 10.1055/s-0031-1284400 21922445

[17] Brotis AG Tasiou A Paterakis K Tzerefos C Fountas KN. Complications associated with surgery for thoracic disc herniation: a systematic review and network meta-analysis. World Neurosurg. (2019) 132:334–42. 10.1016/j.wneu.2019.08.202 31493617

[18] McInerney J Ball PA. The pathophysiology of thoracic disc disease. Neurosurg Focus. (2000) 9(4):e1. 10.3171/foc.2000.9.4.2 16833239

[19] Lesoin F Rousseaux M Autricque A Reesaul Y Villette L Clarisse J. Thoracic disc herniations: evolution in the approach and indications. Acta Neurochir (Wien). (1986) 80(1-2):30–4. 10.1007/BF01809554 3706011

[20] Hulme A. The surgical approach to thoracic intervertebral disc protrusions. J Neurol Neurosurg Psychiatry. (1960) 23(2):133–7. 10.1136/jnnp.23.2.133 14403949 PMC495343

[21] Gibson RDS Wagner R Gibson JNA. Full endoscopic surgery for thoracic pathology: an assessment of supportive evidence. EFORT Open Rev. (2021) 6(1):50–60. 10.1302/2058-5241.6.200080 33532086 PMC7845567

[22] Kasliwal MK. Evolution and current status of surgical management of thoracic disc herniation—a review. Clin Neurol Neurosurg. (2024) 236:108055. 10.1016/j.clineuro.2023.108055 37992532

[23] Fiani B Siddiqi I Reardon T Sarhadi K Newhouse A Gilliland B. Thoracic endoscopic spine surgery: a comprehensive review. Int J Spine Surg. (2020) 14(5):762–71. 10.14444/7109 33046537 PMC7671455

[24] Snyder LA Smith ZA Dahdaleh NS Fessler RG. Minimally invasive treatment of thoracic disc herniations. Neurosurg Clin N Am. (2014) 25(2):271–7. 10.1016/j.nec.2013.12.006 24703446

[25] Wang YB Chen SL Cao C Zhang K Liu LM Gao YZ. Percutaneous transforaminal endoscopic discectomy and fenestration discectomy to treat posterior ring apophyseal fractures: a retrospective cohort study. Orthop Surg. (2020) 12(4):1092–9. 10.1111/os.12698 32583556 PMC7454149

[26] Liu W Li Q Li Z Chen L Tian D Jing J. Clinical efficacy of percutaneous transforaminal endoscopic discectomy in treating adolescent lumbar disc herniation. Medicine (Baltimore). (2019) 98(9):e14682. 10.1097/MD.0000000000014682 30817599 PMC6831190

[27] Liang J Lian L Liang S Zhao H Shu G Chao J. Efficacy and complications of unilateral biportal endoscopic spinal surgery for lumbar spinal stenosis: a meta-analysis and systematic review. World Neurosurg. (2022) 159:e91–102. 10.1016/j.wneu.2021.12.005 34890849

[28] Choi DJ Choi CM Jung JT Lee SJ Kim YS. Learning curve associated with complications in biportal endoscopic spinal surgery: challenges and strategies. Asian Spine J. (2016) 10(4):624–9. 10.4184/asj.2016.10.4.624 27559440 PMC4995243

